# Transcranial direct current stimulation for post-COVID fatigue: a randomized, double-blind, controlled pilot study

**DOI:** 10.1093/braincomms/fcad117

**Published:** 2023-04-10

**Authors:** Silvia Oliver-Mas, Cristina Delgado-Alonso, Alfonso Delgado-Álvarez, María Díez-Cirarda, Constanza Cuevas, Lucía Fernández-Romero, Andreu Matias-Guiu, María Valles-Salgado, Lidia Gil-Martínez, María José Gil-Moreno, Miguel Yus, Jorge Matias-Guiu, Jordi A Matias-Guiu

**Affiliations:** Department of Neurology, Hospital Clínico San Carlos, San Carlos Health Research Institute (IdISCC), Universidad Complutense de Madrid, 28040 Madrid, Spain; Department of Neurology, Hospital Clínico San Carlos, San Carlos Health Research Institute (IdISCC), Universidad Complutense de Madrid, 28040 Madrid, Spain; Department of Neurology, Hospital Clínico San Carlos, San Carlos Health Research Institute (IdISCC), Universidad Complutense de Madrid, 28040 Madrid, Spain; Department of Neurology, Hospital Clínico San Carlos, San Carlos Health Research Institute (IdISCC), Universidad Complutense de Madrid, 28040 Madrid, Spain; Department of Neurology, Hospital Clínico San Carlos, San Carlos Health Research Institute (IdISCC), Universidad Complutense de Madrid, 28040 Madrid, Spain; Department of Neurology, Hospital Clínico San Carlos, San Carlos Health Research Institute (IdISCC), Universidad Complutense de Madrid, 28040 Madrid, Spain; Department of Neurology, Hospital Clínico San Carlos, San Carlos Health Research Institute (IdISCC), Universidad Complutense de Madrid, 28040 Madrid, Spain; Department of Neurology, Hospital Clínico San Carlos, San Carlos Health Research Institute (IdISCC), Universidad Complutense de Madrid, 28040 Madrid, Spain; Department of Radiology, Hospital Clínico San Carlos, San Carlos Health Research Institute (IdISCC), Universidad Complutense de Madrid, 28040 Madrid, Spain; Department of Neurology, Hospital Clínico San Carlos, San Carlos Health Research Institute (IdISCC), Universidad Complutense de Madrid, 28040 Madrid, Spain; Department of Radiology, Hospital Clínico San Carlos, San Carlos Health Research Institute (IdISCC), Universidad Complutense de Madrid, 28040 Madrid, Spain; Department of Neurology, Hospital Clínico San Carlos, San Carlos Health Research Institute (IdISCC), Universidad Complutense de Madrid, 28040 Madrid, Spain; Department of Neurology, Hospital Clínico San Carlos, San Carlos Health Research Institute (IdISCC), Universidad Complutense de Madrid, 28040 Madrid, Spain

**Keywords:** COVID-19, post-COVID syndrome, brain stimulation, fatigue, cognitive

## Abstract

Fatigue is one of the most frequent and disabling symptoms of the post-COVID syndrome. In this study, we aimed to assess the effects of transcranial direct current stimulation on fatigue severity in a group of patients with post-COVID syndrome and chronic fatigue. We conducted a double-blind, parallel-group, sham-controlled study to evaluate the short-term effects of anodal transcranial direct current stimulation (2 mA, 20 min/day) on the left dorsolateral prefrontal cortex. The modified fatigue impact scale score was used as the primary endpoint. Secondary endpoints included cognition (Stroop test), depressive symptoms (Beck depression inventory) and quality of life (EuroQol-5D). Patients received eight sessions of transcranial direct current stimulation and were evaluated at baseline, immediately after the last session, and one month later. Forty-seven patients were enrolled (23 in the active treatment group and 24 in the sham treatment group); the mean age was 45.66 ± 9.49 years, and 37 (78.72%) were women. The mean progression time since the acute infection was 20.68 ± 6.34 months. Active transcranial direct current stimulation was associated with a statistically significant improvement in physical fatigue at the end of treatment and 1 month as compared with sham stimulation. No significant effect was detected for cognitive fatigue. In terms of secondary outcomes, active transcranial direct current stimulation was associated with an improvement in depressive symptoms at the end of treatment. The treatment had no effects on the quality of life. All the adverse events reported were mild and transient, with no differences between the active stimulation and sham stimulation groups. In conclusion, our results suggest that transcranial direct current stimulation on the dorsolateral prefrontal cortex may improve physical fatigue. Further studies are needed to confirm these findings and optimize stimulation protocols.

## Teaser

In a randomized, double-blind, parallel-group and sham-controlled study, Oliver-Mas *et al*. find that eight sessions of anodal transcranial direct current stimulation on the left prefrontal dorsolateral cortex improve physical fatigue in patients with the post-COVID syndrome, with no adverse events.

## Graphical Abstract

**Graphical Abstract:**
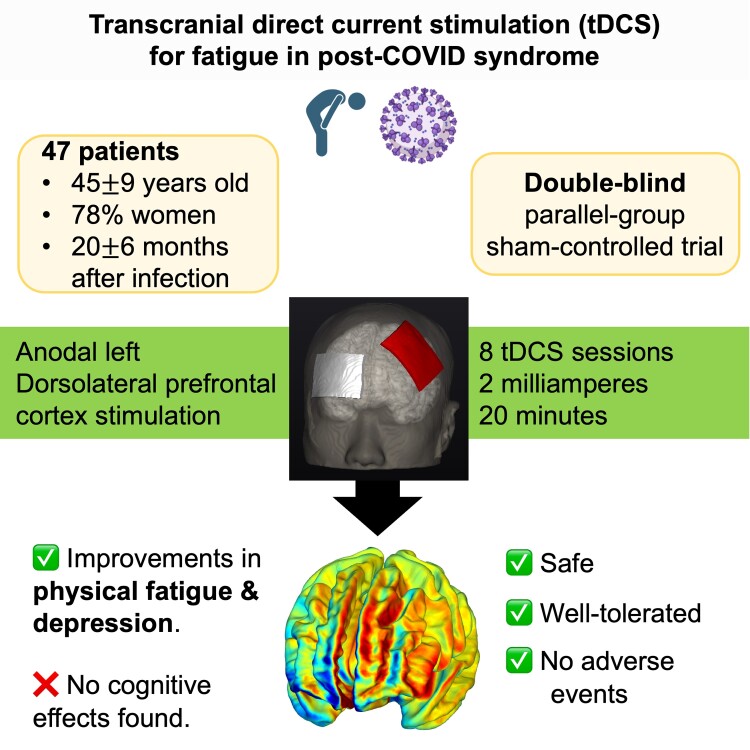

## Introduction

Post-COVID syndrome (or long COVID) is a new disorder occurring in people with a history of SARS-CoV-2 infection that presents with symptoms persisting for at least three months after the acute onset of coronavirus disease 2019 (COVID-19).^1^ Although many symptoms have been described in patients with this condition, fatigue and cognitive dysfunction are among the most frequent and disabling.^2^

Fatigue is defined as a feeling of tiredness and lack of energy that has a negative impact on daily living activities.^3,4^ In post-COVID syndrome, fatigue is present at rest and is also usually triggered with effort, often manifesting with some delay. Persistent fatigue is more frequent in women and has not been associated with the severity of acute illness. Fatigue usually has a physical and a cognitive dimension, which may have different pathophysiological mechanisms.^5,6^ The detection of brain damage in neuroimaging studies in patients with post-COVID syndrome and in histopathological studies in individuals who died due to COVID-19 supports the hypothesis of central nervous system dysfunction in patients with this condition.^7-10^ Furthermore, research with transcranial magnetic stimulation has found connectivity changes potentially associated with gamma-aminobutyric acid (GABA)-ergic neurotransmission dysfunction.^11-2^

Management of fatigue is generally challenging, and effective treatments are lacking.^13^ Transcranial direct current stimulation (tDCS) is a safe, portable, and non-invasive neuromodulation technique.^14^ Previous studies have described the positive effects of non-invasive brain stimulation on fatigue associated with other disorders, such as multiple sclerosis, Parkinson’s disease, stroke, or autoimmune disorders.^15-18^ Conversely, in other studies, clinical efficacy results were inconclusive.^19^ Although the mechanisms associated with fatigue may be different across disorders, a brain network comprising the striatum, the dorsolateral prefrontal cortex, the dorsal anterior cingulate, the ventromedial prefrontal cortex and the anterior insula has been associated with fatigue in several disorders.^20,21^ Alterations have been identified in connectivity between these regions and others, generally showing reduced connectivity between these nodes and other frontal regions and increased connectivity with more posterior regions.^21^ This may suggest that neuromodulation of these networks could have a potential effect on fatigue severity. In patients with COVID-19, the first studies have shown positive effects on persistent olfactory dysfunction and respiratory rehabilitation for critically ill patients.^22,23^ In addition, three case reports have found positive effects in post-COVID syndrome targeting the left dorsolateral prefrontal cortex.^24,25^ This target has also been used for brain stimulation in other causes of fatigue, because this region has been involved in the pathophysiology of physical and cognitive fatigue, and the stimulation could modulate key corticostriatal networks associated with fatigue.^26-28^

Thus, we hypothesized that stimulation of the left dorsolateral prefrontal cortex could modulate fatigue severity in patients with the post-COVID syndrome. We aimed to assess the effects of tDCS on fatigue severity in a group of patients with the post-COVID syndrome and presenting chronic fatigue. We conducted a double-blind, parallel-group, sham-controlled study to evaluate the short-term effects of tDCS on the dorsolateral prefrontal cortex. As secondary outcomes, we evaluated changes in quality of life, depressive symptoms and cognition.

## Materials and methods

### Design

**Figure 1:**
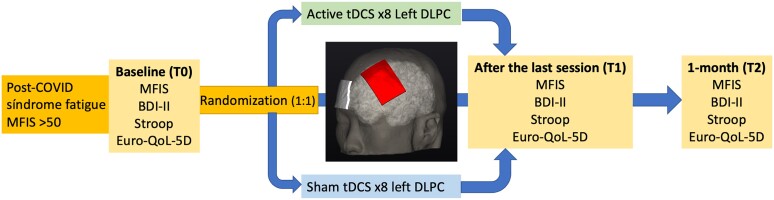
**Summary of the study design**. Patients were assessed at baseline (T0) with MFIS, BDI-II, Stroop test and Euro-QoL-5D and randomized to receive eight sessions of either active tDCS or sham tDCS. Patients were assessed again with the same scales after the last session (T1) and after 1-month (T2). BDI-II, Beck depression inventory; DLPC, left dorsolateral prefrontal cortex; MFIS, modified fatigue impact scale; tDCS, transcranial direct current stimulation.

This study is a double-blind, parallel-group, sham-controlled pilot study conducted at the Department of Neurology of Hospital Clínico San Carlos, in Madrid, from April to July 2022. The study was approved by the local Ethics Committee (code 22/120-E) and conducted according to the Declaration of Helsinki and its later amendments. The protocol was registered on Clinicaltrials.gov (STIMULATE-COVID, NCT05252481) before the onset of the study. All participants gave informed consent. Figure 1 summarizes the study design.

### Participants

We gathered patients with post-COVID syndrome who met the following inclusion criteria and none of the exclusion criteria:

- Inclusion criteria:
  - Diagnosis of COVID-19 with positive RT-PCR results at least 6 months before inclusion in the study
  - Diagnosis of post-COVID syndrome according to the World Health Organization criteria.^1^
  - Symptoms of fatigue (e.g. lack of energy) reported by the patient. Fatigue is temporally associated with SARS-CoV-2 infection and is of sufficient severity to interfere with activities of daily living [modified fatigue impact scale (MFIS) > 50].
- Exclusion criteria:
  - Diagnosis of any neurological, psychiatric or medical disorder with potential impact on fatigue (e.g. multiple sclerosis, anaemia, chronic kidney disease, cancer and fibromyalgia).
  - History of traumatic brain injury or central nervous system infection before COVID-19
  - Radiotherapy or chemotherapy for cancer
  - Severe sensory deficits
  - Use of drugs or presence of uncontrolled medical conditions with a potential impact on fatigue
  - History of substance abuse, including alcohol
  - Any contraindication for tDCS (metallic implants, brain devices, pacemakers and head injury).

### Randomization and blinding

Patients were randomly allocated at a 1:1 ratio into two groups using a web-based random number generator (https://www.studyrandomizer.com/). Simple randomization was used, with no stratification. Both patients and assessors were blinded to the allocation. The researcher performing the tDCS sessions was also responsible for patient enrolment and had no access to assessment results. The group in which participants were allocated was not revealed until the last participant completed the last assessment of the study.

### Transcranial direct current stimulation protocol

The stimulation protocol consisted of eight sessions for eight consecutive days over the course of two consecutive weeks, with a break over the weekend between weeks. Stimulation was performed using a Nurostym tES device (Neuro Device Group S.A.; Poland). The treatment group received active tDCS using sponge-covered electrodes soaked in saline solution. Electroconductive gel was added in some cases when necessary to reduce the impedance. The anode (electrode size 5 × 7 cm) was placed over the left dorsolateral prefrontal cortex (F3, according to the international 10–20 system). The target was localized with neuronavigation using a standard template and the BrainSight neuronavigation system (Rogue Research Inc.; Canada). The cathode (size 5 × 7 cm) was placed on the contralateral supraorbital region. The current was ramped up for 15 s until reaching 2 mA and ramped down for 15 s at the end of the stimulation period. A constant current of 2 mA intensity was delivered for 20 min. In the sham group, electrodes were placed in the same regions; in this case, the current was ramped up and down at the beginning and the end of the session, but it was turned off during the 20 min session. Impedance values were constantly checked during the stimulation and were kept at <10 kΩ. Patients were treated in the morning (8.30 am–12 am) or in the afternoon (12 am–4 pm), according to the availability of patients.

### Clinical evaluation and endpoints

Patients were assessed at baseline (T0), immediately after the last stimulation session (T1), and one month after treatment completion (T2). A baseline examination was conducted the same day or the day before the first session of tDCS.

The primary outcome was a change in fatigue, as assessed by the MFIS.^29,30^ The MFIS consists of 21 items and evaluates three dimensions: MFIS-physical (scores 0–36), MFIS-cognitive (0–40) and MFIS-psychosocial (0–8). Each item is scored on a five-point Likert-type scale from 0 (no fatigue) to 4 (severe fatigue). The total MFIS score is the sum of the scores on the three subscales and ranges from 0 (no fatigue) to 84 (the most severe fatigue).

The secondary outcomes were:

- Changes in cognition. We used the Stroop Color-Word Interference test,^31^ as previous works by our research group have found that this test is the tool that most frequently detects abnormal results in patients with post-COVID syndrome and is best correlated with fatigue severity.^6,32^ The Stroop test is a measure of cognitive flexibility, selective attention, inhibition, and processing speed. It comprises three tasks: (i) reading the names of colours printed in blank ink (W); (ii) naming different colour patches (C); and (iii) naming the colour of the ink in colour words printed in an incongruent colour ink (e.g. ‘blue’ is printed in red ink and the patient must read ‘red’) (CW). The final score is the number of correctly named items in 45 s. We used the interference score proposed by Golden^31^ (IG), which is calculated using the following formula:
  IG=CW–[(W×C)/(W+C)]
- Changes in depressive symptoms. These were evaluated with the Beck depression inventory (BDI-II), which contains 21 items, scored on a four-point scale from 0 (no symptoms) to 3 (severe symptoms), which assess the severity of depressive symptoms in adults. The total score ranges from 0 to 63.^33^
- Changes in quality of life. This was assessed with the EuroQol-5D, the recommended preference-based measure in the NICE guidance on methods for health technology assessment.^34^ This scale assesses five dimensions of health: mobility, self-care, usual activities, pain/discomfort and anxiety/depression. Each item has three possible answers according to the level of function, ranging from 1 (no problems) to 3 (disabling problems). We used the visual analogue scale (VAS), depicting a straight vertical line where 0 indicates a worst imaginable state of health and 100 indicates a best imaginable state of health. The participant must mark the point on the vertical line that best reflects, in their opinion, their overall health status on the day of data collection.^35^

At baseline, participants were also evaluated with the state-trait anxiety inventory (STAI). For illustrative purposes, an STAI-S score ≥40 was considered to indicate clinically significant anxiety, and a BDI-II score ≥19 was interpreted as moderate or severe depression. Patients were also evaluated with the symbol digit modalities test (SDMT) (written version, 90 seconds).^36^

### Sample size

At the time of the study conception, no reliable information was available about the frequency of fatigue in post-COVID syndrome. However, in other disorders, a 10-point change in the MFIS is considered clinically significant.^37^ Assuming a type I error of 0.05, a power of 80%, a standard deviation of 10 for the main outcome variable, and a drop-out rate of 10%, the sample size was established as 40 participants (20 per group).

### Electric field simulation

In a subgroup of 37 patients, MRI was available for electric field simulations.^38^ Images were acquired on a 3 T Magnet (GE Signa Architect) and a 48-channel head coil. 3D T1-weighted images were acquired in a sagittal MPRAGE sequence with the following parameters: number of slices = 200, slice thickness = 1 mm, field of view 256 mm, matrix = 256 × 256, flip angle = 8, preparation time = 974 ms, recovery time = 700 ms, TR = 7.7 ms, TE = 3.1 ms, NEX = 1 and acquisition time = 9:27. The software SimNIBS (version 4.0.0) running in Matlab (version 2019a) was used to estimate the electric field induced by the protocol of tDCS used in our study.^39^ First, head models were created using the ‘charm’ pipeline for every patient. Second, we performed simulations for each patient according to our protocol. Specifically, we used a montage with anodal F3 (intensity +2 mA) and cathodal FP2 (−2 mA) (electrode characteristics: rectangular shape, 7 × 5 cm, thickness = 5, saline-soaked sponges) according to the international 10–20 EEG system. Conductivity was set at the default parameters of SimNIBS. The electric field strength and focality were extracted for each participant. Strength was considered as the 99.0th of the field magnitudes and expressed as V/m. Focality was measured as the grey matter volume with an electric field greater or equal to 75% of the peak value (99.9th percentile). Then, we calculated the mean and standard deviation of the normal component of the electric field for the whole sample in FsAverage space.

### Statistical analysis

Descriptive data are shown as either mean ± standard deviation or as absolute frequency (percentage). The Shapiro–Wilk test was used for normality testing. Baseline clinical and demographic characteristics were compared using the χ^2^ test or the *t-*tests, as appropriate.

Repeated measures ANOVA was conducted for each outcome using ‘time’ (T0, T1, T2) as a within-subjects factor and ‘group’ (active versus sham tDCS) as a between-subjects factor. The ‘time-by-group’ interaction was estimated. Data were log-transformed due to the non-normality of total and subscale MFIS scores in the sample and to reduce the influence of atypical values. In addition, the following covariates or factors were introduced: sex, months since clinical onset, baseline anxiety (STAI-S) and baseline depression (BDI-II). These variables were introduced due to the statistically significant differences observed between the active and sham groups in terms of sex and months since clinical onset and the well-known influence of anxiety and depression on fatigue scores.^40^ For the analysis of the Stroop effect, years of education were also added to the model. Mauchly’s test was used to determine whether the assumption of sphericity was met, and Levene’s test to assess the homogeneity of variance. When a violation of sphericity was detected, Greenhouse–Geisser corrected *P*-values were reported. *Post hoc* analyses were performed for statistically significant main effects or interactions, using *P*-values adjusted according to the Holm–Bonferroni method and considering three comparisons for the main effect (time) and 15 for the time-by-group interaction. Effect sizes were evaluated with Cohen’s *d*, which compares differences between two means. Effects were regarded as small for *d* = 0.2, moderate for *d* = 0.5 and large for *d* = 0.8. Statistical significance was set at *P* < 0.05.

In line with the previous literature, a clinically meaningful change was defined as a reduction of at least 10 points in MFIS-total scores.^37^ For the BDI-II, a change of at least three points is considered to indicate a clinically significant change in depressive symptoms.^41^ The percentage of patients reaching this cut-off for each treatment group was calculated. We also calculated the percentage of agreement between patients reaching this cut-off at T1 and T2.

### Data availability

The data that support the findings of this study are available from the corresponding author, upon reasonable request.

## Results

### Participants

**Figure 2:**
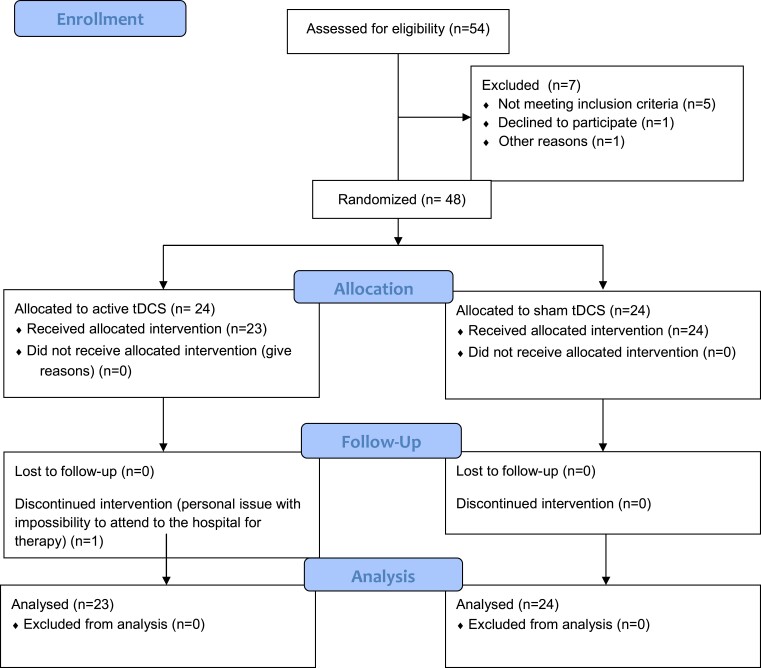
**Study flow diagram**. Flow diagram showing participant flow through each stage of the trial.

Fifty-four participants were initially selected. Seven were considered ineligible for the following reasons: previous diagnosis of fibromyalgia (1), already receiving non-invasive brain stimulation (1), and absence of significant fatigue (one after a telephone interview and three due to low MFIS scores after assessment). One patient declined to participate in the study after the baseline assessment due to extreme fatigue with the impossibility to attend the sessions. Therefore, 48 patients were randomly assigned to receive either active tDCS or sham tDCS (Fig. 2). One patient in the active tDCS group discontinued intervention after the first session due to personal issues. Thus, the final sample size for analysis was 47 participants. The mean age was 45.66 ± 9.49 years, and 37 (78.72%) were women. The mean time since the acute infection was 20.68 ± 6.34 months. The baseline demographic and clinical characteristics of our sample are summarized in Tables 1 and 2. At baseline, depression was detected in 20 participants (42.55%) and anxiety in 30 (63.82%). Fifteen patients (65.2%) of the active group and 16 (66.7%) of the sham were treated in the morning (*X*^2^ = 0.011, *P* = 0.917).

**Table 1:** Demographic characteristics

	Active tDCS	Sham tDCS	*t* or χ^2^ (*P*-value)
Number of patients	23	24	-
Age	47.26 ± 9.05	44.12 ± 9.83	1.13 (0.262)
Sex (women)	15 (65.21%)	22 (91.66%)	4.90 (**0.027**)
Months since acute SARS-CoV-2 infection	18.65 ± 7.30	22.62 ± 4.63	−2.23 (**0.030**)
Years of education	16.52 ± 2.82	15.91 ± 2.84	0.73 (0.468)
Handedness	100% right	100% right	-
Arterial hypertension	4 (17.39%)	2 (8.33%)	0.86 (0.352)
Diabetes mellitus	1 (4.34%)	1 (4.16%)	0.00 (0.975)
Dyslipidemia	5 (21.73%)	7 (29.16%)	0.34 (0.559)
Smoking	0 (0%)	2 (8.33%)	2.00 (0.157)
Hospitalization due to acute COVID-19	4 (17.4%)	3 (12.5%)	0.22 (0.638)
ICU admission due to acute COVID-19	1 (4.3%)	1 (4.2%)	0.001 (0.975)

*P*-values **in bold** indicate statistically significant differences. Values are shown as number of patients (percentage) (N, %) or mean ± standard deviation. ICU, intensive care unit; N, Number of patients; tDCS, transcranial direct current stimulation.

**Table 2:** Baseline clinical variables

	Active tDCS	Sham tDCS	*t* or χ^2^ (*P*-value)
MFIS (total score)	68.26 ± 10.15	70.12 ± 8.06	−0.69 (0.488)
MFIS-physical	29.65 ± 5.07	31.41 ± 3.12	−1.44 (0.156)
MFIS-cognitive	32.39 ± 4.98	32.29 ± 6.36	0.06 (0.953)
MFIS-psychosocial	6.47 ± 1.50	6.41 ± 1.76	0.12 (0.898)
BDI-II	19.26 ± 8.56	19.58 ± 11.26	−0.11 (0.913)
Depression	11 (47.82%)	9 (37.50%)	0.51 (0.474)
STAI-state	45.73 ± 11.72	41.45 ± 12.43	1.21 (0.231)
STAI-trait	49.17 ± 11.18	49.91 ± 12.82	−0.21 (0.834)
EuroQoL-5D (VAS)	49.56 ± 14.61	47.50 ± 12.85	0.515 (0.609)
Stroop trial 1	79.56 ± 25.17	84.95 ± 28.07	−1.640 (0.108)
Stroop trial 2	55.65 ± 15.38	60.04 ± 20.82	−1.779 (0.082)
Stroop trial 3	33.78 ± 11.02	38.16 ± 14.97	−1.474 (0.148)
IG (Stroop interference)	1.53 ± 10.70	3.10 ± 7.78	−0.578 (0.566)
SDMT	31.08 ± 10.56	34.70 ± 11.62	−1.11 (0.270)

Values are shown as a number of patients (percentage) (N, %) or mean ± standard deviation.

BDI-II, Beck depression inventory; MFIS, modified fatigue impact scale; SDMT, symbol digit modalities test; STAI, state-trait anxiety inventory; tDCS, transcranial direct current stimulation; VAS, visual analogue scale.

### Primary outcome

**Figure 3:**
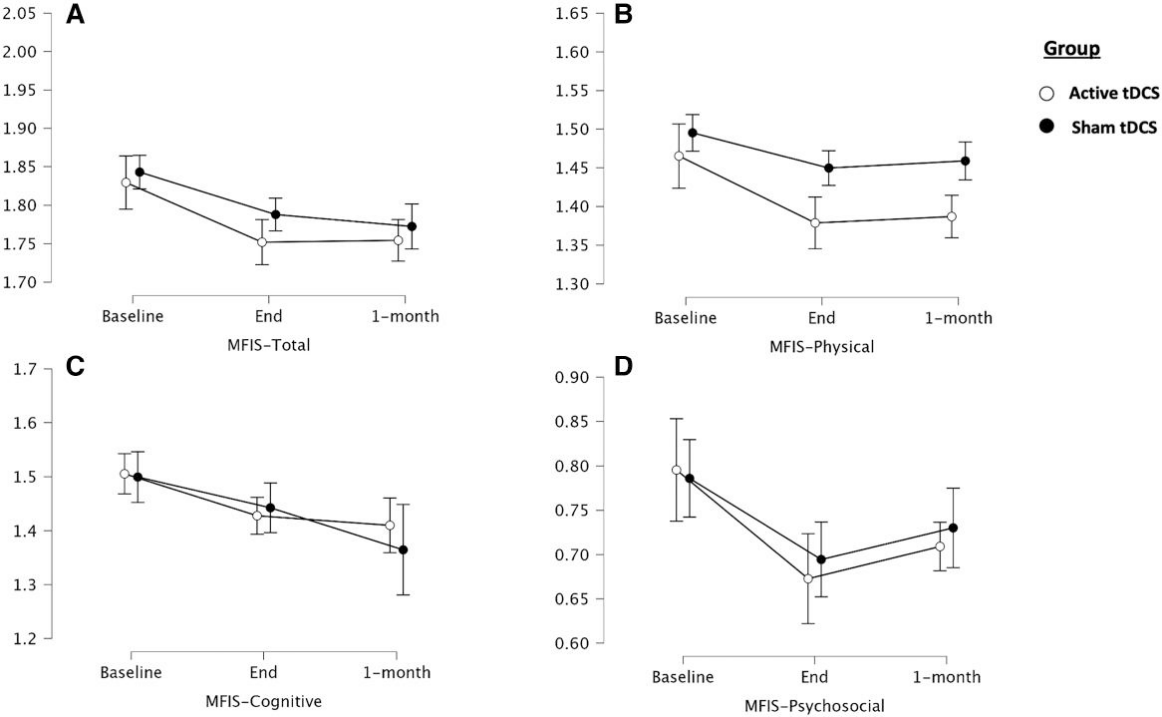
**Fatigue scores over time**. Plot representing changes (means with 95% confidence intervals) in study outcomes (*n* = 47). (**A**) MFIS-total; (**B**) MFIS-physical; (**C**) MFIS-cognitive; (**D**) MFIS-psychosocial. Y-axis of each plot represents the log-transformed values. MFIS, modified fatigue impact scale; tDCS, transcranial direct current stimulation.

For MFIS-total, time-by-group interaction effect was not significant (F_(2,82)_ = 1.730, *P* = 0.184). A significant main effect of time was observed (F_(2,82)_ = 3.420, *P* = 0.037). *Post hoc* comparisons revealed a statistically significant difference between T0 and T1 (mean difference of 0.067, *t* = 4.263, *P* < 0.001) and between T0 and T2 (mean difference of 0.074, *t* = 4.699, *P* < 0.001), but not between T1 and T2 (mean difference of 0.007, *t* = 0.435, *P* = 0.664) (Table 3, Fig. 3A).

**Table 3:** Repeated measures ANOVA and *post hoc* analysis for primary and secondary outcomes

Outcome variable		Within-subjects effects		Between-subjects effect	*Post hoc* analysis (time)			*Post hoc* analysis (time × group)					
		Time	Time × group interaction	Group	T0 versus T1	T0 versus T2	T1 versus T2	Active T0 versus Active T1	Active T0 versus Active T2	Sham T0 versus Sham T1	Sham T0 versus Sham T2	Active T1 versus Sham T1	Active T2 versus Sham T2
MFIS-total	F or T (*P*-value)	3.420 (0.037)	1.730 (0.184)	3.638 (0.064)	4.263 (<0.001)	4.699 (<0.001)	0.435 (0.664)	4.800 (<0.001)	4.397 (<0.001)	1.765 (0.569)	2.707 (0.091)	−2.531 (0.130)	−1.381 (0.852)
MFIS-physical	F or T (*P*-value)	2.940 (0.058)	3.517 (0.034)	5.307 (0.026)	4.063 (<0.001)	0.549 (0.002)	−0.668 (0.506)	5.144 (<0.001)	4.150 (0.001)	1.198 (1.000)	1.127 (1.000)	−2.907 (0.052)	−2.363 (0.188)
MFIS-cognitive	F or T (*P*-value)	1.447 (0.240)	0.55 (0.496)	0.079 (0.781)	1.890 (0.125)	3.523 (0.002)	1.633 (0.125)	2.232 (0.313)	2.924 (0.067)	0.694 (1.000)	2.348 (0.277)	−0.985 (1.000)	0.148 (1.000)
MFIS-psychosocial	F or T (*P*-value)	1.197 (0.307)	0.667 (0.516)	1.927 (0.172)	4.445 (<0.001)	2.532 (0.026)	−1.913 (0.059)	4.420 (<0.001)	2.581 (0.151)	2.338 (0.240)	1.279 (1.000)	−1.650 (0.823)	−1.260 (1.000)
Stroop (IG)	F or T (*P*-value)	0.422 (0.657)	0.116 (0.891)	1.697 (0.200)	−0.250 (0.934)	−1.019 (0.945)	−0.769 (0.939)	−0.434 (1.000)	−0.696 (1.000)	0.029 (1.000)	−0.792 (1.000)	−1.398 (1.000)	−1.235 (1.000)
BDI-II	F or T (*P*-value)	2.042 (0.136)	3.447 (0.036)	0.935 (0.339)	3.057 (0.006)	5.022 (<0.001)	1.965 (0.053)	3.607 (0.007)	3.275 (0.020)	1.144 (1.000)	4.115 (0.001)	−1.934 (0.517)	0.092 (1.000)
EuroQoL-5D (VAS)	F or T (*P*-value)	4.324 (0.016)	2.172 (0.120)	0.228 (0.636)	−1.718 (0.109)	−3.667 (0.001)	−1.950 (0.109)	−1.763 (0.816)	−1.840 (0.763)	−0.886 (1.000)	−3.326 (0.020)	1.355 (1.000)	−0.428 (1.000)

In each cell, F- or T-statistic (*P*-values) are shown.

For MFIS-physical, there was a significant time-by-group interaction effect (F_(2,82)_ = 3.517, *P* = 0.034) and a trend towards statistical significance for the main effect of time (F_(2,82)_ = 2.940, *P* = 0.058). A significant main effect of the group was found (F_(1,41)_ = 5.307, *P* = 0.026). *Post hoc* analysis revealed differences between T0 and T1 (mean difference of 0.105, *t* = 5.144, *P* < 0.001, *d* = 1.032) and between T0 and T2 (mean difference of 0.085, *t* = 4.150, *P* = 0.001, *d* = 0.833) for the active group, but not for the sham group (T0 versus T1: mean difference of 0.029, *t* = 1.198, *P* = 1.0, *d* = 0.282; T0 versus T2 = 0.027, *t* = 1.127, *P* = 1.0, d = 0.266). No statistically significant differences were observed between T1 and T2 for the active treatment group (mean difference of −0.020, *t* = −0.994, *P* = 1.0) (Fig. 3B).

For MFIS-cognitive and MFIS-psychosocial, no statistically significant interaction or main effects were observed (Fig. 3C and D).

There were no statistically significant intergroup differences in the percentage of patients showing a clinically significant change in MFIS-total at the end of treatment (34.8% in the active group versus 45.8% in the sham group, χ^2^ = 0.596, *P* = 0.440) and at one month (43.5% versus 45.8%, χ^2^ = 0.026, *P* = 0.871).

When comparing the patients with clinically significant changes in fatigue at T1 and T2, 65.9% remained in the same category for MFIS-total.

### Secondary outcomes

**Figure 4:**
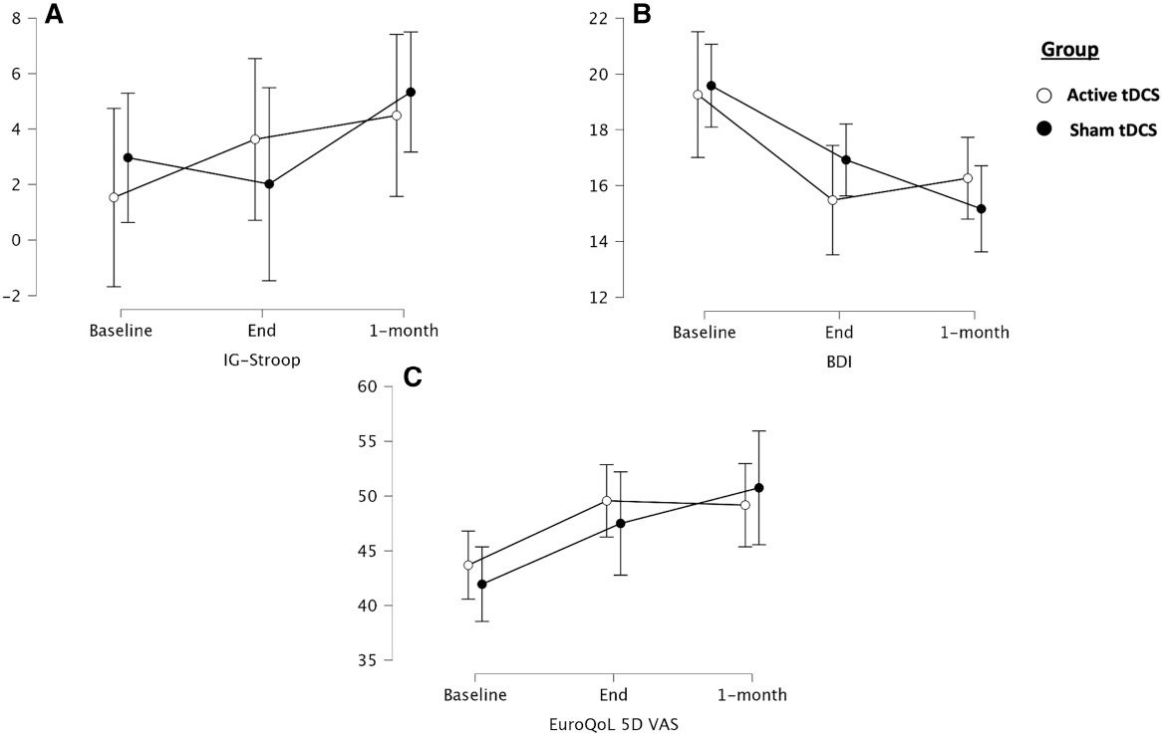
**Secondary outcomes over time**. Plot representing changes (means with 95% confidence intervals) in study secondary outcomes (*n* = 47). (**A**) Stroop (interference score, IG); (**B**) BDI-II; (**C**) EuroQoL-5D (VAS). Y-axis represents the interference score (A) of the Stroop test, the raw score of BDI-II (B) and Euro-QoL-5D (VAS) (C). BDI, Beck depression inventory-II; tDCS, transcranial direct current stimulation; IG: index of golden (Stroop Interference).

We found no statistically significant interaction between Stroop test results and the group, and there were no main effects for group or time (Fig. 4A).

Regarding depression, a statistically significant time-by-group interaction was found (F_(2,84)_ = 3.447, *P* = 0.036), but no main effect of time was found (F_(2,84)_ = 2.042, *P* = 0.136). *Post hoc* analysis revealed differences between T0 and T1 for active tDCS (mean difference of 4.184, *t* = 3.607, *P* = 0.007, *d* = 0.625), but not for sham tDCS (mean difference of 1.572, *t* = 1.144, *P* = 1.0, *d* = 0.235). We also found significant differences between T0 and T2 in both the active tDCS (mean difference of 3.799, *t* = 3.275, *P* = 0.020, *d* = 0.567) and the sham tDCS groups (mean difference of 5.656, *t* = 4.115, *P* = 0.001, *d* = 0.844) (Fig. 4B). At T1, 47.8% of the active group and 54.2% of the sham group showed a clinically significant change, with no statistically significant differences (χ^2^ = 0.189, *P* = 0.664). At T2, the percentage of patients showing a clinically significant change compared with baseline was greater in the sham group, with a trend towards statistical significance (66.7% versus 39.1%, χ^2^ = 3.57, *P* = 0.059). When comparing the patients with clinically significant changes at T1 and T2, 76.6% remained in the same category.

The analysis of quality of life with the EuroQol-5D showed no statistically significant time-by-group interaction (F_(2,84)_ = 2.172, *P* = 0.120). However, a statistically significant main effect of time was detected (F_(2,84)_ = 4.324, *P* = 0.016) (Fig. 4C). All results are shown in Table 3 and Supplementary Table 1.

### Adverse events

Four patients (17.39%) in the active stimulation group and five patients (20.83%) in the sham stimulation group reported adverse events (χ^2^ = 0.09, *P* = 0.764): headache (three in the active group and four in the sham group) and dizziness (one in each group). Adverse events were regarded as mild in all cases. No severe adverse events were observed.

### Blinding integrity

After the first tDCS session, 15 (65.21%) of participants in the active tDCS group and 16 (66.66%) in the sham tDCS group believed that they had received real stimulation (χ^2^ = 0.01, *P* = 0.917). After the last session, 13 (56.52%) and 11 (45.83%) of participants in the active and sham groups, respectively, believed that they had received real stimulation (χ^2^ = 0.53, *P* = 0.464).

### Electric field simulation

**Figure 5:**
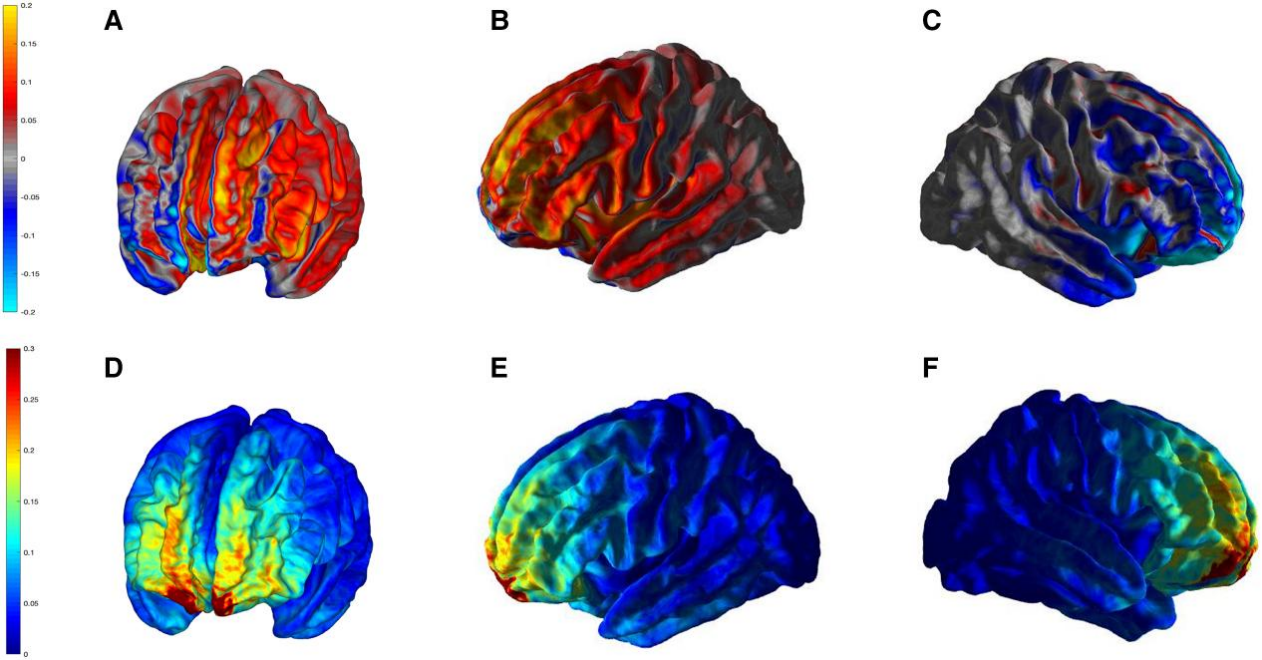
**Electric field calculations**. It was conducted on the 37 participants in which MRI was available. Values are shown in V/m. (**A-C**) mean of the normal field component simulations; (**D-F**) standard deviation of the normal field component simulations. 3D-rendered images of the front left (A and D), lateral left (B and E) and lateral right (C and F).

The simulation of the electric field for the 37 participants in which MRI was available showed a mean field strength of 0.44 ± 0.18 V/m and a focality of 6.89 ± 4.06 mm^3^ (Fig. 5). The maximum electric field strength was in the left prefrontal cortex, especially in the superior frontal gyrus.

## Discussion

In this study, we aimed to evaluate the effect of tDCS on fatigue in patients with the post-COVID syndrome. The most interesting finding was a positive effect on physical fatigue with active stimulation as compared to sham stimulation, according to MFIS-physical scores, after controlling for several potential confounding factors (depression, anxiety, sex and months since clinical onset). Furthermore, a general improvement was observed in MFIS-total scores, which was more pronounced in the active tDCS group; this group presented a mean reduction of 9.8 points in MFIS-total score at the end of treatment, compared with 7.7 points in patients receiving sham stimulation. However, the time-by-group interaction for the MFIS-total score was not statistically significant. Overall, these findings suggest that anodal tDCS applied over the left dorsolateral prefrontal cortex has a positive effect in patients with the post-COVID syndrome; to our knowledge, tDCS is one of the first therapeutic options to show positive results in this condition.

Conversely, we did not find a positive effect of tDCS on MFIS-cognitive or Stroop test scores. Although some studies suggest that dorsolateral prefrontal tDCS could enhance cognitive performance, especially attention function and working memory, recent meta-analyses have shown conflicting results.^42,43^ In a recent study into post-COVID syndrome, we found that the Stroop test was the test that most strongly correlated with subjective fatigue,^6^ although this test is also associated with other cognitive functions. Thus, our results suggest that short-term prefrontal tDCS has no significant effects on cognitive fatigue as measured by subjective and objective tests. This finding comes as no surprise due to the action mechanism of tDCS, which does not directly induce cortical activity.^14^ In this regard, the combination of cognitive training and tDCS could be useful and should be examined in future studies. Furthermore, studies specifically focused on the cognitive effects of tDCS should include a more comprehensive neuropsychological assessment. As we only performed the Stroop test to evaluate cognitive changes, effects on other neuropsychological tests or functions cannot be excluded. Time of day is another factor that should be considered when evaluating the cognitive effects of prefrontal stimulation because early morning could be more optimal to stimulate episodic memory retrieval, especially in younger adults.^44^ Regarding psychosocial fatigue, we did not observe significant changes. However, this finding is of uncertain clinical significance because, according to recent studies, the psychosocial subscale of the MFIS has a low factor loading in exploratory factor analysis of MFIS, as well as low reliability, and should not be interpreted independently of the other subscales.^45^

Regarding the secondary outcomes, we only detected a statistically significant effect in depression. Specifically, patients receiving active tDCS showed improvements in depressive symptoms after treatment compared with those receiving sham stimulation. This is consistent with previous studies, as tDCS has a grade B recommendation for major depression.^46^ Surprisingly, at one month, patients in the sham group also showed an improvement in depression. Given the time elapsed between treatment completion and the final assessment, this change may be explained by spontaneous improvements in a subgroup of patients.^47^ No changes were observed in the quality of life after eight tDCS sessions. Although fatigue has a large impact on quality of life, this finding is consistent with previous studies into other disorders, which have also found no improvements in quality of life with short-term therapies for fatigue.^17^

Another interesting aspect of our study is that 34% of patients changed from showing a clinically significant change to showing no significant changes, or vice versa. The definition of a clinically significant response was drawn from studies into other disorders; however, longitudinal studies should seek to specifically define this parameter for the post-COVID syndrome. In any case, the percentage of patients who changed from one category to another confirms the fluctuating nature of fatigue.^48^

Regarding safety and tolerability, tDCS was well-tolerated, with only minimal adverse events, regardless of the allocation group. Treatment adherence was good, in line with previous reports of the use of tDCS in other disorders.^46^ In this study, we applied eight sessions of tDCS, based on previous research experience using tDCS for other causes of fatigue, such as multiple sclerosis, fibromyalgia and chronic fatigue syndrome, in which studies generally used at least five sessions of treatment.^49,50^ However, future studies are needed to determine the optimal number of sessions to treat this disorder.

According to the estimated electric field models, the protocol used in this study obtained the maximal electric field strength over the left prefrontal cortex, especially in the left superior and middle frontal gyri. These regions are connected with other nodes of the cognitive control network, default mode network, and motor control network.^51^ In addition, previous studies have evidenced that applying tDCS over the prefrontal cortex induces subcortical changes and modulates the dopaminergic system.^27^ Overall, this suggests that tDCS would be able to modulate the main cortico-striatal networks previously associated with fatigue in healthy people and different diseases,^52-57^ and our study provides preliminary evidence of the possibility of regulating these processes in patients with the post-COVID syndrome.

Our study has some limitations. First, participants were not stratified by sex, with the sham stimulation group including a higher percentage of women. However, the placebo effect is generally more frequent in women,^58^ and ANOVA models controlled for sex. Second, the effect of tDCS on other variables was not analyzed. For instance, some patients spontaneously reported improvements in sleep quality. Sleep disturbances are frequent in post-COVID syndrome, and the potential of tDCS for improving sleep disturbances and sleep quality has already been evaluated by some authors;^59^ this improvement may in turn result in a reduction in fatigue. Third, the target selection and protocol were based on evidence from other disorders. In this regard, other target locations (e.g. M1) or longer protocols may be more appropriate for fatigue or other symptoms associated with post-COVID syndrome or in other contexts associated with COVID-19 that may benefit from tDCS.^60^ For instance, a recent study showed an increase in the number of ventilator-free days after high-definition tDCS combined with respiratory rehabilitation.^23^ Greater understanding of brain network alterations in post-COVID syndrome and the pathophysiology of this syndrome may help to identify patient subgroups, with a view to developing optimized protocols and individualized brain stimulation parameters.^61,62^

In conclusion, our study confirms the safety of tDCS in post-COVID syndrome and suggests a potential benefit of left dorsolateral prefrontal cortex stimulation for physical fatigue. However, this is a pilot study, and our results should, therefore, be considered preliminary. Further research should confirm these findings in larger samples and optimize the brain stimulation protocols to maximize the effects of this therapy.

## Supplementary Material

fcad117_Supplementary_Data

## Abbreviations

BDI-II =: beck depression inventory
COVID-19 =: coronavirus disease 19
MFIS =: modified fatigue impact scale
MPRAGE =: magnetization prepared—rapid gradient echo
RT-PCR =: reverse transcription polymerase chain reaction
SARS =: severe acute respiratory syndrome
SDMT =: symbol digit modalities test
STAI =: state-trait anxiety inventory
tDCS =: transcranial direct current stimulation
tES =: transcranial electrical stimulation
