# Supplementary material for: Transcranial direct current stimulation for post-COVID fatigue: a randomized, double-blind, controlled pilot study
Source: Brain Commun. 2023 Apr 10;5(2):fcad117. doi: 10.1093/braincomms/fcad117 (PMC10116605; doi:10.1093/braincomms/fcad117)
Supplement: fcad117_Supplementary_Data [file fcad117_supplementary_data.docx]

| Supplementary Table 1. Descriptive values (mean and standard deviation) of each outcome at T0, T1, and T2. | | | | | | |
| --- | --- | --- | --- | --- | --- | --- |
|  | T0 | | T1 | | T2 | |
| Outcome variable | Active tDCS | Sham tDCS | Active tDCS | Sham tDCS | Active tDCS | Sham tDCS |
| MFIS-total | 68.26±10.15 | 70.12±8.06 | 58.47±13.56 | 62.41±11.39 | 58.78±14.58 | 60.50±12.69 |
| MFIS-physical | 29.65±5.07 | 31.41±3.12 | 25.39±7.52 | 28.58±4.87 | 25.73±7.85 | 29.08±4.39 |
| MFIS-cognitive | 32.39±4.98 | 32.29±6.36 | 27.60±6.56 | 28.41±6.29 | 27.21±25.79 | 25.79±9.08 |
| MFIS-psychosocial | 6.47±1.50 | 6.41±1.76 | 5.08±1.72 | 5.41±1.97 | 5.39±1.67 | 5.62±2.06 |
| Stroop (IG) | 1.53±10.70 | 3.10±7.78 | 3.62±8.90 | 2.44±9.08 | 4.49±10.23 | 5.33±6.81 |
| BDI-II | 19.26±8.56 | 19.58±11.26 | 15.47±6.70 | 16.91±10.37 | 16.26±6.71 | 15.16±10.17 |
| EuroQoL-5D (VAS) | 49.56±14.61 | 47.50±12.85 | 49.56±14.61 | 47.50±12.85 | 49.17±16.99 | 50.75±15.27 |
